# Sporadic Creutzfeldt-Jakob Disease and Other Proteinopathies in Comorbidity

**DOI:** 10.3389/fneur.2020.596108

**Published:** 2020-11-30

**Authors:** Eva Parobkova, Julie van der Zee, Lubina Dillen, Christine Van Broeckhoven, Robert Rusina, Radoslav Matej

**Affiliations:** ^1^Department of Pathology and Molecular Medicine, Third Faculty of Medicine, Charles University and Thomayer Hospital, Prague, Czechia; ^2^National Reference Laboratory for Human Prion Diseases, Thomayer Hospital, Prague, Czechia; ^3^Neurodegenerative Brain Diseases Group, VIB Center for Molecular Neurology, Vlaams Instituut voor Biotechnologie (VIB), Antwerp, Belgium; ^4^Department of Biomedical Sciences, University of Antwerp, Antwerp, Belgium; ^5^Department of Neurology, Third Faculty of Medicine, Charles University and Thomayer Hospital, Prague, Czechia; ^6^Department of Pathology, First Faculty of Medicine, Charles University and General University Hospital, Prague, Czechia; ^7^Department of Pathology, Third Faculty of Medicine, Charles University and Kralovske Vinohrady University Hospital, Prague, Czechia

**Keywords:** Creutzfeldt-Jakob disease, Alzheimer's disease, β amyloid, tau protein, neurodegenerative disease

## Abstract

**Background:** Sporadic Creutzfeldt–Jakob disease (sCJD) is the most common type of a group of transmissible spongiform encephalopathies (prion diseases). The etiology of the sporadic form of CJD is still unclear. sCJD can occur in combination with other neurodegenerative diseases, which further complicates the diagnosis. Alzheimer's disease (AD), e.g., is often seen in conjunction with sCJD.

**Method:** In this study, we performed a systematic analysis of 15 genes related to the most important neurodegenerative diseases - AD, frontotemporal dementia, amyotrophic lateral sclerosis, prion disease, and Parkinson's disease - in a cohort of sCJD and sCJD in comorbidity with AD and primary age-related proteinopathy (PART). A total of 30 neuropathologically verified cases of sCJD with and without additional proteinopathies were included in the study. In addition, we compared microtubule-associated protein tau *(MAPT)* haplotypes between sCJD patients and patients with sCJD and PART or sCJD and AD. Then we studied the interaction between the Apolipoprotein E gene (*APOE)* and *PRNP* in sCJD patients.

**Results:** We did not find any causal mutations in the neurodegenerative disease genes. We did detect a p.E318G missense variant of uncertain significance (VUS) in *PSEN1* in three patients. In *PRNP*, we also found a previously described non-pathogenic insertion (p.P84_Q91Q).

**Conclusion:** Our pilot study failed to find any critical differences between pure sCJD and sCJD in conjunction with other comorbid neurodegenerative diseases. Further investigations are needed to better understand this phenomenon.

## Introduction

Neurodegenerative diseases are characterized by intra-or extracellular accumulation of specific protein aggregates in the central nervous system (CNS) (1). These proteins have a predominantly β-sheet form and are found in a number of neurodegenerative diseases such as Alzheimer's disease (AD); synucleinopathies (Parkinson's disease (PD), multiple system atrophy, dementia with Lewy bodies); transmissible spongiform encephalopathies (TSE; also known as prion disease); amyotrophic lateral sclerosis and frontotemporal dementia (2). There is a significant overlap of symptoms resulting from the multiplication and tissue storage of protein aggregates in the brain, leading to progressive neuronal dysfunction and neurodegeneration (3, 4).

Creutzfeldt-Jakob disease (CJD; MIM #176640), the most common human prion disease with an estimated incidence of 2 cases per million per year, is comprised of several clinical-pathological phenotypes and occurs in four unique forms (sporadic, genetic, variant, or acquired), each with seemingly distinct etiologies (5).

CJD can coexist with other neurodegenerative diseases because the presence of both Aβ and tau pathology is not unusual in sporadic and genetic CJD brains (6–9). Primary age-related proteinopathy (PART) is a common pathology involving misfolded tau protein aggregates associated with human aging (10). PART can cause cognitive impairment in the absence of AD (11); additionally, the coexistence of PART and sporadic CJD (sCJD) has been reported (12). A major genetic risk factor for PART is the haplotype of the microtubule-associated protein tau (*MAPT*) (13). The frequency of Apolipoprotein E (APOE) ε4 is much lower in PART, being ~10% (10, 14), whereas its prevalence in AD exceeds 45% (15, 16). These studies suggest that APOE ε4 allele deficiency – in contrast to AD – is not a risk factor for PART.

Coexistence with other neurodegenerations is relatively common in sCJD patients. Since clinical symptoms of sCJD can overlap with manifestations of other comorbid disorders, establishing a clinical diagnosis in patients with rapidly progressive dementia is very difficult (17), and a definite diagnosis can only be made after a neuropathological examination of the brain.

Our goal was to identify disease-associated variants using genetic studies of sCJD patients. For this reason, we compared sCJD patients without any comorbid proteinopathies to sCJD patients with AD and sCJD patients with PART.

## Materials and Methods

### Study Population

Our study was designed as a retrospective study. We included patients with post-mortem confirmed sCJD, as well as information regarding clinical presentation and data from neuropsychological testing, biochemical analysis, EEG, and neuroimaging. Neuropathological diagnoses, including prion protein immunoassays, were provided according to standard protocols National CJD Research & Surveillance Unit. Protocol: Surveillance of CJD in the UK) (18) used by the National reference laboratory for human prion diseases at the Department of Pathology and Molecular Medicine, Prague, Czech Republic. Molecular genetic analyses were performed in the Neurodegenerative Brain Disease group of the VIB Center for Molecular Neurology, Antwerp, Belgium.

We divided our cohort into three subgroups: (1) isolated sCJD neuropathology, (2) sCJD and PART or early stage AD (NIA consensus criteria level “low”) (19), and (3) sCJD with more advanced AD (NIA consensus criteria level A2 and or higher).

### The Molecular Diagnostics Study Group

All autopsied patients (30/30) fulfilled the WHO diagnostic criteria for definite sporadic CJD (18)^1^ and were genetically profiled for the most common genes (*n* = 15) associated with AD (*APP, PSEN1, PSEN2, APOE*), the FTD-ALS spectrum (*MAPT, GRN, TARDBP, FUS, SOD1, VCP*), prion disease *(PRNP*), and PD (*LRRK2, PRKN, SNCA*) (Supplementary Table 1).

Other available clinical data, which were designated as variables (including age at onset, age at death, gender, and symptoms occurring during the disease), were analyzed to determine how they affected the pathogenesis of sCJD and the concomitant Aβ and tau pathologies.

### Genetic Screen

Mutation analyses by gene panel sequencing were performed on genomic DNA extracted from bone marrow. The targeted gene panel captured all exons of the 15 genes and flanking intronic regions to cover the splice sites. Using amplicon target amplification technology (Agilent, https://www.agilent.com), primers were designed using mPCR software (20) (Supplementary Material). Specific target regions were amplified using multiplex PCR, followed by purification of the equimolar pooled amplicons using Agencourt AMPureXP beads (Beckman Coulter, CA, USA). Individual barcodes (Illumina Nextera XT) were incorporated in a universal PCR step prior to sample pooling. Libraries were sequenced on a MiSeq platform using the v3 reagent kit with a paired-end read length of 300 bp (Illumina, San Diego, CA, USA). Non-sense, splice site, indel, and missense variants, with a minor allele frequency (MAF) ≤ 1%, were selected.

### Results

We analyzed data from 30 patients (*n* = 30) with a mean age at onset (AAO) of 58.4 ± 5 years, and a male-to-female ratio of 18:12. Ten cases had sCJD without any other comorbid proteinopathy, 10 cases had sCJD with tauopathy and/or early evolved AD, and 10 cases had sCJD with more developed AD. Family histories were available in 24 cases (82%), with only one patient (3.4%) having a positive family history for dementia. No family histories of CJD were reported. Effects of rare (MAF ≤ 1%) missense variants on protein structure and function were predicted using SIFT (http://sift.jcvi.org/), Polyphen2 (http://genetics.bwh.harvard.edu/pph2/) and SNP&GO (https://snps-and-go.biocomp.unibo.it/snps-and-go/) (Supplementary Table 2).

Clinical manifestations included mild to moderate dementia with predominant executive and speech/language impairment (aphasia, dysarthria) with less impaired memory and visuospatial function. Behavioral and psychiatric manifestations (depression, apathy, irritability, anxiety, aggression, visual hallucinations, and insomnia) were described in most patients. Motor symptoms typically included Parkinsonism, spasticity, gait disturbance, and/or immobility (Supplementary Table 3).

### Mutation Screening

Gene panel screening (15 genes) for variants and mutations associated with AD, FTD-ALS, and PD, revealed 4 rare, protein-modifying variants (Supplementary Table 2). Effects on protein structure and function were predicted using SIFT (http://sift.jcvi.org/), Polyphen2 (http://genetics.bwh.harvard.edu/pph2/) and SNP&GO (https://snps-and-go.biocomp.unibo.it/snps-and-go/). In *PSEN1*, the p.E318G polymorphism in *PSEN1* was found in three patients (10.3%), of which one had pure sCJD, and two were sCJD + AD. No relevant variants were observed in the other AD genes *APP* and *PSEN2*. In *PRNP*, the p.P84_Q91Q insertion was detected in one patient with β-amyloidopathy. This variant is considered non-pathogenic (21). Furthermore, one benign missense variant was present in *GRN* and one in *SOD1*. We did not find any potential disease-causing mutations in the PD genes *SNCA, LRRK2*, and *PRKN;* silent mutations were found (Supplementary Table 2).

#### Genetic Predisposing Factors - ε4 Allele of Apolipoprotein *E* (*APOE*)

**Figure 1:**
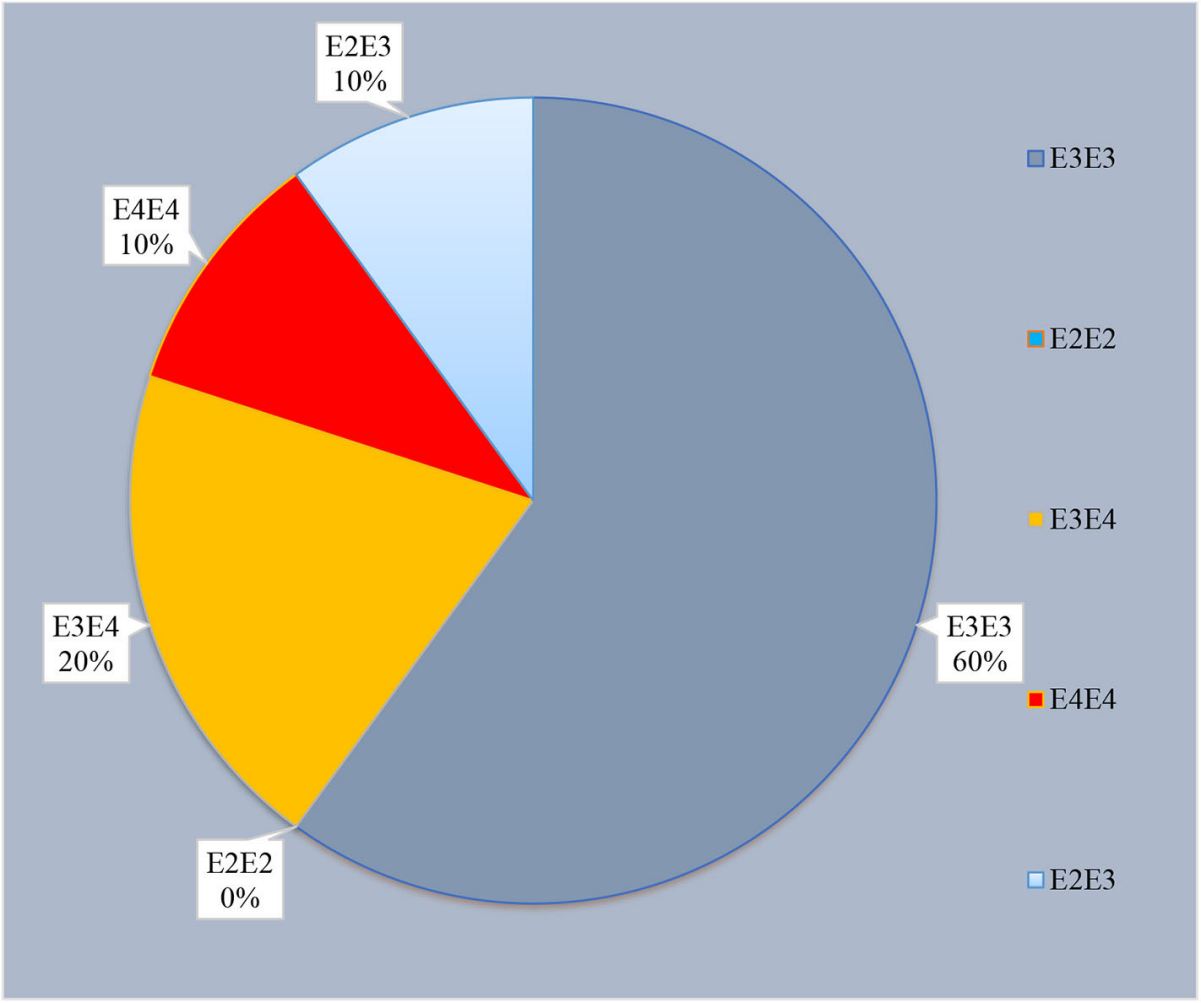
Distribution of *APOE* polymorphism carriers in our cohort (*n* = 30).

*APOE* polymorphic variants were tested at codons 112 and 158. Of the 30 cases in our study, 10% (*n* = 3) carried the ε4 allele of the *APOE* gene (Figure 1). All three cases were AD level A2 (AAO 62, 75, 83 years). The distribution of the polymorphic codon 129 of *PRNP* and *APO* genotypes in sCJD patients are shown in Supplementary Table 4. We found no association between *APOE* ε4 allele status and sCJD; however, the *APOE* ε4 was seen in two *PRNP* M129M homozygotes (*n* = 2).

### MAPT Haplotype Association With Sporadic CJD

We analyzed MAPT haplotypes in both isolated sCJD cases and in cases with sCJD and tauopathy. We identified only one case with the H2/H2 haplotype, and they were in the comorbid subgroup (Supplementary Table 5). As such, our study shows no evidence of an association between MAPT gene variations and sCJD, which could have contributed to the tau deposits in the CNS.

## Discussion

In our study of 30 cases of sCJD in the Czech Republic (the annual rate of definite CJD is about 20 cases/yr.), we analyzed the most important genes related to neurodegeneration. The cognitive profile in our patients was characterized by a heterogeneous manifestation, with predominant involvement of executive and speech/language functions with a significant proportion also having behavioral manifestations (including visual hallucinations).

We did not detect any pathogenic mutations in the *PRNP* gene. Our study also tried to determine if there were any predisposing genetic factors that could account for the occurrence of comorbid Aβ and tau protein deposits in CJD brains. Previous studies have provided evidence that comorbid proteinopathy is not unusual in CJD brains, although the exact mechanism by which β-amyloid and tau deposits spread within brain tissue remains unclear (22). Since several studies have documented a possible spread of β-amyloid in brain tissue (23, 24), we performed a mutation analysis of *APP* (Aβ encoding exons) as well as the coding region of *PSEN1* and *PSEN2*. However, we did not find any mutations in the genes that would explain the increased Aβ42 production.

There is only sparse evidence supporting the potential interaction between *APOE* and *PRNP* in sCJD. Recent studies that analyzed the influence of *APOE* on CJD have yielded discordant results. Three of our cases had the *APOE* ε4 genotype (AAO > 70 years on average), i.e., β-amyloidopathy level A2 and Methionine/Methionine homozygosity at codon 129 of the *PRNP* gene (M129M). Recent studies have suggested variants of PRNP129 (methionine/methionine, methionine/valine, valine/valine) as possible modifiers of AD disease (25). However, because of the small sample size of our study, this interpretation should be approached cautiously. Further studies should be carried out to assess the effects of PRNP129 in the AD phenotype. We found no influence of the *APOE* genotype relative to the age at onset, nor any significant differences in the distribution of the *APOE* ε4 and ε2 genotypes relative to those with isolated sCJD and those with sCJD and AD. Our results are consistent with other studies showing that *APOE* is not a risk factor for CJD (26–29).

The pathology of tau in sCJD brains is not unique, and in our cohort, this additional pathology was seen in 6 of the 30 definite CJD patients (30%) (6). Tau is encoded by the *MAPT* gene, and there are two common *MAPT* extended haplotypes, i.e., H1 and H2 (29). Only one study has investigated the role of MAPT in the etiology of sCJD (30). There is somewhat more evidence regarding the role of MAPT haplotypes (H1 and H2) in neurodegenerative diseases. H1 has been linked to FTLD and AD (31), whereas H2 is associated with a lower risk for developing late-onset AD (32). Our study shows no evidence for any association between *MAPT* gene haplotypes and sCJD.

The coexistence of CJD and PD is exceedingly rare. Several reported case studies show that α-synuclein amyloid deposits in CJD patients are associated with a slower disease course. The precise molecular mechanism explaining how misfolded α-synuclein accumulates and spreads in synucleinopathies is still unknown (33). Sequence or copy number variants in at least six genes (*SNCA, LRRK2, PRKN, PINK1, DJ-1*, and *ATP13A2*) have been identified to cause monogenic forms of PD (34). To date, no mutations responsible for PD have been reported in patients with CJD. Due to the low incidence of patients with proven CJD and PD, it is not clear whether there are gene interactions between CJD and PD. Our study, however, was not focused on the issue of sCJD and synucleinopathy, due to the extremely low incidence of both pathologies in comorbidity. This issue is, nevertheless, a promising direction for future research, and as such, it could help us better understand the genetic background as well as perhaps offer novel therapeutic options.

In conclusion, we failed to find any association between the investigated genes and the accumulation of specific protein aggregates in the examined brain tissue. These findings suggest that comorbid neurodegenerative disorders in sCJD behave as if they were independent processes taking place within the same brain; additionally, the underlying pathophysiology of comorbid protein deposits in CJD appears to have a complex multifactorial origin.

It would, however, be promising in the future to examine other risk genes for AD, FTD, and PD, and their potential association with CJD (Supplementary Table 6) (35). The search for genetic evidence of clinical, pathological, and possible molecular overlap between neurodegenerative diseases certainly needs to continue and would be best done with a larger multicenter cohort.

## Data Availability Statement

The data analyzed in this study is subject to the following licenses/restrictions: This manuscript utilizes proprietary data. Requests to access these datasets should be directed to Julie van der Zee, julie.vanderzee@uantwerpen.vib.be, Neurodegenerative Brain Diseases Group, VIB Center for Molecular Neurology, VIB, Antwerp, Belgium. Primers for the targeted assay were designed using proprietary mPCR software from Agilent (previously Multiplicom) (20).

## Ethics Statement

Ethical review and approval was not required for the study on human participants in accordance with the local legislation and institutional requirements. Written informed consent from the participants' legal guardian/next of kin was not required to participate in this study in accordance with the national legislation and the institutional requirements.

## Author Contributions

JZ created the concept and design of the project. CVB provided laboratory facilities for the practical implementation of the entire project, provided complete instrumentation of the VIB Center for Molecular Neurology. JZ and CVB critically revised the manuscript. LD provided recommendations and specific approaches to the samples analysis (analysis tools) as an expert technician in the laboratory. RR diagnosed in detail the neurological cases mentioned in the article. RM verified the neuropathological diagnosis of neurodegenerations and approved the final version to be published. All authors participated in the manuscript.

## Conflict of Interest

The authors declare that the research was conducted in the absence of any commercial or financial relationships that could be construed as a potential conflict of interest.

## Footnotes

^1^www.cjdsupport.org/, https://www.cjdsupport.org.au/site/wp-content/uploads/2017/04/PRNP-Guidelines-160417.pdf
