# Supplementary material for: Sporadic Creutzfeldt-Jakob Disease and Other Proteinopathies in Comorbidity
Source: Front Neurol. 2020 Nov 30;11:596108. doi: 10.3389/fneur.2020.596108 (PMC7735378; doi:10.3389/fneur.2020.596108)
Supplement: Supplementary file 1 [file Table_1.docx]

Table S1-Gene content of the targeted assay

| Gene |  | Associated phenotype | Locus (GRCh38.p12) |
| --- | --- | --- | --- |
| *APP* |  | AD | chr21:25,880,550-26,170,770 |
| *PSEN1* |  | AD | chr14:73,136,507-73,223,691 |
| *PSEN2* |  | AD | chr1: 226,870,616-226,896,098 |
| *APOE* |  | AD | chr19: 44,905,796-44,909,393 |
| *PRNP* |  | Prion D | chr20: 4,686,350-4,701,590 |
| *GRN* |  | FTD | chr17: 44,345,246-44,353,106 |
| *C9orf72* |  | FTD-ALS | chr9: 27,546,546-27,573,481 |
| *MAPT* |  | FTD | chr17: 45,894,554-46,028,334 |
| *VCP* |  | FTD-ALS | chr9: 35,056,064-35,072,627 |
| *TARDBP* |  | FTD-ALS | chr1: 11,012,344-11,025,739 |
| *FUS* |  | ALS | chr16: 31,180,139-31,191,605 |
| *SOD1* |  | ALS | chr21: 31,659,666-31,668,931 |
| *LRRK2* |  | PD | chr12: 40,196,744-40,369,285 |
| *PRKN* |  | PD | chr6: 161,347,417-162,727,775 |
| *SNCA* |  | PD | chr4: 89,724,099-89,837,161 |

### Abbreviations

APP: Amyloid precursor protein, PSEN1: Presenilin 1, PSEN2: Presenilin 2, APOE: Apolipoprotein E, PRNP: Prion protein, GRN: Progranulin, C9orf72: [Chromosome 9](https://en.wikipedia.org/wiki/Chromosome_9) [open reading frame](https://en.wikipedia.org/wiki/Open_reading_frame) 72, MAPT: Microtubule-associated protein tau, VCP: Valosin-containing protein, TARDBP: Transactive response DNA binding protein, FUS: Fused In Sarcoma, SOD1: Superoxide dismutase 1, LRRK2: Leucine-rich repeat kinase 2, PRKN: Parkin, SNCA: Synuclein Alpha. AD: Alzheimer's disease, Prion D: Prion diseases, FTD: F[rontotemporal dementia](https://en.wikipedia.org/wiki/Frontotemporal_dementia), FTD-ALS: [Frontotemporal dementia](https://en.wikipedia.org/wiki/Frontotemporal_dementia) and [amyotrophic lateral sclerosis](https://en.wikipedia.org/wiki/Amyotrophic_lateral_sclerosis), ALS: [Amyotrophic lateral sclerosis](https://en.wikipedia.org/wiki/Amyotrophic_lateral_sclerosis), PD: Parkinson's disease.
