# Supplementary material for: Sporadic Creutzfeldt-Jakob Disease and Other Proteinopathies in Comorbidity
Source: Front Neurol. 2020 Nov 30;11:596108. doi: 10.3389/fneur.2020.596108 (PMC7735378; doi:10.3389/fneur.2020.596108)
Supplement: Supplementary file 2 [file Table_2.docx]

Table S2-Observerd variants in genes associated with AD (APP, PSEN1, PSEN2, APOE) and the FTD-ALS spectrum (MAPT, TARDBP, GRN, FUS, SOD1, VCP), and PD (LRRK2, PRKN, SNCA)

| gene | region | genomic mutation | predicted protein | ref SNP ID | frequency | polyphen-2 | SIFT | SNP&GO |
| --- | --- | --- | --- | --- | --- | --- | --- | --- |
| *PSEN1* | exon9 | c.953A>G | p.E318G | rs17125721 | 3/30 | benign | tolerated | neutral |
| *GRN* | exon11 | c.1297C>T | p.R433W | rs63750412 | 1/30 | benign |  |  |
| *SOD1* | exon 4 | c.272A>C | p.D91A | rs80265967 | 1/30 | benign | tolerated | neutral |

Deletion

| gene | region | genomic mutation | predicted protein | ref SNP ID | frequency |
| --- | --- | --- | --- | --- | --- |
| *PRNP* | exon2 | c.204_227del | p.P84_Q91 | rs1389957 | 1/30 |

Frameshift mutations

| gene | region | genomic mutation | predicted protein | ref SNP ID | frequency |
| --- | --- | --- | --- | --- | --- |
| *LRRK2* | exon 34 | c.4915del | p.R1639Gfs*14 | rs756089224 | 15/30 |
| *PRKN* | exon11 | c.1283del | p.N428Mfs*6 |  | 22/30 |

Silent variations

| gene | region | genomic mutation | predicted protein | ref SNP ID | frequency |
| --- | --- | --- | --- | --- | --- |
| *PRNP* | exon2 | c.351A>G | p.A117A | RS8124214 | 1/30 |
| *PSEN2* | exon4 | c.69T>C | p.A23A | rs11405 | 26/30 |
|  | exon4 | c.129C>T | p.N43N | rs6759 | 24/30 |
|  | exon5 | c.261C>T | p.H87H | rs1046240 | 24/30 |
|  | exon6 | c.414C>T | p.S138S | rs747738607 | 1/30 |
| *MAPT* | exon9 | c.1479G>A | p.P493P | rs1052551 | 8/30 |
|  | exon10 | c.1632A>G | p.A544A | rs1052553 | 8/30 |
|  | exon10 | c.1716T>C | p.N572N | rs17652121 | 8/30 |
|  | exon10 | c.1761G>A | p.P587P | rs1568305 | 8/30 |
| *GRN* | exon5 | c.384T>C | p.D128D | rs25646 | 1/30 |
| *FUS* | exon3 | c.147C>A | p.G49G | rs781810 | 11/30 |
|  | exon4 | c.291C>T | p.Y97Y | rs76570520 | 20/30 |
|  | exon12 | c.1197T>G | p.G399G | rs76570520 | 5/30 |
| *LRRK2* | exon10 | c.1104G>A | p.E368E | rs1243256303 | 13/30 |

## Observed variants in 30 pathology-confirmed CJD patients. For the rare variants with MAF ≤ 1%, in silico modeling of pathogenicity was performed using SIFT (http://sift.jcvi.org/), Polyphen2 (http://genetics.bwh.harvard.edu/pph2/), and SNP&GO (https://snps-and-go.biocomp.unibo.it/snps-and-go/).
