# Supplementary material for: Sporadic Creutzfeldt-Jakob Disease and Other Proteinopathies in Comorbidity
Source: Front Neurol. 2020 Nov 30;11:596108. doi: 10.3389/fneur.2020.596108 (PMC7735378; doi:10.3389/fneur.2020.596108)
Supplement: Supplementary file 3 [file Table_3.docx]

## Table S3-Clinical presentation of sCJD cases

|  | Age of onset | Disease duration (month) | Family history | Pathology | Biochemical subtype | PRNP (SNP 129) | Neuropathological diagnosis PART  ARTAG  AD | Ataxia | Disorientations | Sensitive disorders | Cognitive deficits | Walking instability | Extrapyramidal signs | Phatic disorders | Tremor | Visual symptoms | Myoclonus | Word disorders | Dementia | Behavioral disorders |
| --- | --- | --- | --- | --- | --- | --- | --- | --- | --- | --- | --- | --- | --- | --- | --- | --- | --- | --- | --- | --- |
| NP1 | 60-70 | 1 | S | PPR,AD | 1 | MM | sCJD, A2B1C2 | x | x |  | x | x |  |  |  | x | x |  | x |  |
| NP2 | 60-70 | 2 | S | PPR, AD | 1 | MM | sCJD, A2B2C1 |  | x |  |  | x |  |  |  |  |  | x | x |  |
| NP3 | 60-70 | 2 | S | PPR, AD | 1 | MM | SCJD, A2B2C2 |  | x |  | x | x |  |  |  |  |  | x | x |  |
| NP4 | 60-70 | 2 | S | PPR, AD | 1 | VV | sCJD, A2B2C2 | x |  |  | x |  | x | x |  | x |  |  | x |  |
| NP5 | 70-80 | 1 | S | PPR, AD | 1 | MM | sCJD, A2B2C2 |  | x |  | x |  |  | x |  |  |  |  | x |  |
| NP6 | 70-80 | 1.5 | S | PPR, AD | 1 | MM | sCJD, A2B2C2 |  |  |  |  |  |  |  |  | x |  | x | x |  |
| NP7 | 75-85 | 1 | S | PPR, AD | 1 | MM | sCJD, A2B2C2 |  | x | x | x |  | x |  |  |  | x |  | x |  |
| NP8 | 65-75 | 2 | S | PPR, AD | 1 | MM | sCJD, A2B2C2 | x | x |  | x | x |  |  | x | x | x |  | x |  |
| NP9 | 60-70 | 1.5 | S | PPR, AD | 1 | MV | sCJD, A2B2C2 | x | x |  |  |  |  |  |  | x | x |  | x |  |
| NP10 | 60-70 | 6 | S | PPR, AD | 2A | MV | sCJD, A3B2C2 |  |  |  |  |  |  |  |  |  | x |  | x |  |
| NP11 | 60-70 | 2 | S | PPR | 1 | MM | sCJD |  |  |  | x |  |  |  |  |  |  | x | x | x |
| NP12 | 40-50 | 2 | S | PPR | 1 | MV | sCJD |  | x |  |  |  |  |  |  |  | x | x | x | x |
| NP13 | 50-60 | 6 | S | PPR | 1 | VV | sCJD |  |  |  | x | x | x |  |  |  | x |  | x |  |
| NP14 | 60-70 | 2 | S | PPR | 1 | VV | sCJD |  |  |  |  | x |  |  |  |  |  |  | x |  |
| NP15 | 60-70 | 2 | S | PPR | 1 | MM | sCJD |  |  |  | x | x | x | x |  |  | x | x | x |  |
| NP16 | 55-65 | 24 | S | PPR | 2A | MM | sCJD |  | x |  | x |  |  |  |  |  |  |  | x |  |
| NP17 | 70-80 | 2 | S | PPR | 1 | MM | sCJD |  | x | x | x | x |  |  | x | x |  | x | x |  |
| NP18 | 50-60 | 2 | S | PPR | 1 | MM | sCJD |  | x |  | x |  |  |  | x | x | x | x | x |  |
| NP19 | 45-55 | 12 | S | PPR | 2A | MM | sCJD |  | x |  | x |  |  |  |  |  |  |  | x |  |
| NP20 | 40-50 | 4 | S | PPR | 1 | VV | sCJD | x |  |  | x | x | x | x | x | x | x | x | x |  |
| NP21 | 70-80 | 2 | S | PPR, tau | 1 | VV | sCJD, PART | x | x | x | x |  |  |  |  |  |  |  | x |  |
| NP22 | 60-70 | 4 | S | PPR, tau | 1 | MM | sCJD, PART |  |  | x | x | x | x | x |  | x |  |  | x |  |
| NP23 | 50-60 | 10 | S | PPR, tau | 2 | MM | sCJD, PART | x |  |  | x | x | x |  | x | x |  |  | x |  |
| NP24 | 50-60 | 2 | S | PPR, tau | 1 | MV | sCJD, PART |  | x |  | x | x |  |  |  | x |  | x | x |  |
| NP25 | 65-75 | 2 | S | PPR, tau | 1 | MM | sCJD, PART |  |  |  |  |  |  | x |  |  | x |  | x |  |
| NP26 | 80-90 | 1-2 | S | PPR, tau | 1 | MM | sCJD, PART | x |  |  |  | x |  |  |  | x | x |  | x |  |
| NP27 | 65-75 | 9 | S | PPR, AD | 1 | VV | sCJD, A1B1C1 |  |  |  |  | x |  | x |  |  | x | x | x | x |
| NP28 | 60-70 | 10 | S | PPR, AD | 1 | MV | sCJD, A1B1C1 |  |  |  |  |  |  |  |  |  |  |  | x |  |
| NP29 | 60-70 | 5 | S | PPR, AD | 1 | VV | cCJD, A1B1C1 | x | x | x |  | x |  |  |  |  | x |  | x |  |
| NP30 | 60-70 | 6 | S | PPR, AD | 1 | VV | sCJD, A1B2C1 |  |  |  |  | x | x |  |  |  | x | x | x |  |

Symbols: S: sporadic, F: familial, PPR = prionopathy, AD: β-amyloidopathy, tau: tauopathy, M: male, F: female, PART: primary age-related tauopathy, ARTAG: aging-related tau astrogliopathy, MM: methionine/methionine, MV: methionine/valine, VV: valine/valine.
