# Supplementary material for: Sporadic Creutzfeldt-Jakob Disease and Other Proteinopathies in Comorbidity
Source: Front Neurol. 2020 Nov 30;11:596108. doi: 10.3389/fneur.2020.596108 (PMC7735378; doi:10.3389/fneur.2020.596108)
Supplement: Supplementary file 4 [file Table_4.docx]

Table S4-Association between CJD and PRNP gene polymorphism at codon 129 defined by APOE ε4 allele status *sCJD genotypes*

|  | ɛ2/ ɛ2 | ɛ2/ ɛ3 | ɛ2/ ɛ4 | ɛ3/ ɛ3 | ɛ3/ ɛ4 | ɛ4/ ɛ4 |
| --- | --- | --- | --- | --- | --- | --- |
| M129M | 0 | 2 | 0 | 2 | 2 | 0 |
| M129V | 0 | 0 | 0 | 1 | 0 | 0 |
| V129V | 0 | 0 | 0 | 2 | 1 | 0 |

*sCJD and tauopathy and/or early evolved AD*

|  | ɛ2/ ɛ2 | ɛ2/ ɛ3 | ɛ2/ ɛ4 | ɛ3/ ɛ3 | ɛ3/ ɛ4 | ɛ4/ ɛ4 |
| --- | --- | --- | --- | --- | --- | --- |
| M129M | 0 | 1 | 0 | 3 | 0 | 0 |
| M129V | 0 | 0 | 0 | 1 | 1 | 0 |
| V129V | 0 | 0 | 0 | 4 | 0 | 0 |

*sCJD and developed AD*

|  | ɛ2/ ɛ2 | ɛ2/ ɛ3 | ɛ2/ ɛ4 | ɛ3/ ɛ3 | ɛ3/ ɛ4 | ɛ4/ ɛ4 |
| --- | --- | --- | --- | --- | --- | --- |
| M129M | 0 | 0 | 0 | 4 | 1 | 2 |
| M129V | 0 | 1 | 0 | 0 | 0 | 1 |
| V129V | 0 | 0 | 0 | 1 | 0 | 0 |
