# Supplementary material for: Sporadic Creutzfeldt-Jakob Disease and Other Proteinopathies in Comorbidity
Source: Front Neurol. 2020 Nov 30;11:596108. doi: 10.3389/fneur.2020.596108 (PMC7735378; doi:10.3389/fneur.2020.596108)
Supplement: Supplementary file 5 [file Table_5.docx]

## Table S5-Genotype APOE and haplotype MAPT

| dnumber (CMN) | APOE (genotype) | MAPT (haplotype) | PRNP | Neuroptahological diagnosis |
| --- | --- | --- | --- | --- |
| FM8/18 | ɛ3ɛ3 | H1/H1 | negative | sCJD, A2B1C2 |
| MA95/17 | ɛ3ɛ3 | H1/H1 | negative | sCJD, A2B2C1 |
| ČV102/17 | ɛ3ɛ4 | H1/H1 | negative | SCJD, A2B2C2 |
| ŠS1/18 | ɛ3ɛ3 | H1/H2 | negative | sCJD, A2B2C2 |
| ŠP9/19 | ɛ4ɛ4 | H1/H1 | p.P84_Q91 | sCJD, A2B2C2 |
| EA65/19 | ɛ3ɛ3 | H1/H1 | negative | sCJD, A2B2C2 |
| LF48/18 | ɛ4ɛ4 | H1/H1 | negative | sCJD, A2B2C2 |
| KJ30/19 | ɛ3ɛ3 | H1/H2 | negative | sCJD, A2B2C2 |
| PA135/16 | ɛ2ɛ3 | H1/H2 | negative | sCJD, A2B2C2 |
| PJ30/18 | ɛ4ɛ4 | H1/H2 | negative | sCJD, A3B2C2 |
| BP64/17 | ɛ3ɛ3 | H1/H1 | negative | sCJD |
| ZD125/17 | ɛ3ɛ3 | H2/H2 | negative | sCJD |
| LT153/17 | ɛ3ɛ3 | H1/H2 | negative | sCJD |
| BM10/8 | ɛ3ɛ4 | H1/H1 | negative | sCJD |
| AM13/18 | ɛ3ɛ3 | H1/H1 | negative | sCJD |
| ŠJ14/18 | ɛ2ɛ3 | H1/H1 | negative | sCJD |
| VJ47/18 | ɛ2ɛ3 | H1/H2 | negative | sCJD |
| KE139/18 | ɛ3ɛ4 | H1/H2 | negative | sCJD |
| KI33/19 | ɛ3ɛ4 | H1/H1 | negative | sCJD |
| LF44/19 | ɛ3ɛ3 | H1/H1 | negative | sCJD |
| AF112/16 | ɛ3ɛ3 | H1/H1 | negative | sCJD, PART |
| ŠL113/16 | ɛ2ɛ3 | H1/H2 | negative | sCJD, PART |
| HB123/16 | ɛ3ɛ3 | H1/H1 | negative | sCJD, PART |
| FJ15/18 | ɛ3ɛ3 | H1/H1 | negative | sCJD, PART |
| HJ93/18 | ɛ3ɛ3 | H1/H1 | negative | sCJD, PART |
| VJ27/19 | ɛ3ɛ3 | H1/H1 | negative | sCJD, PART |
| KA90/17 | ɛ3ɛ3 | H1/H1 | negative | sCJD, A1B1C1 |
| MF20/18 | ɛ3ɛ4 | H1/H1 | negative | sCJD, A1B1C1 |
| KE66/18 | ɛ3ɛ3 | H1/H2 | negative | sCJD, A1B1C1 |
| SI56/19 | ɛ3ɛ3 | H1/H2 | negative | sCJD, A1B2C1 |

Symbols: sCJD sporadic Creutzfeldt-Jakob disease
