# Supplementary material for: Sporadic Creutzfeldt-Jakob Disease and Other Proteinopathies in Comorbidity
Source: Front Neurol. 2020 Nov 30;11:596108. doi: 10.3389/fneur.2020.596108 (PMC7735378; doi:10.3389/fneur.2020.596108)
Supplement: Supplementary file 6 [file Table_6.docx]

Table S6-List of new genes

| Disease Categories | Candidate Genes Selection |
| --- | --- |
| Alzheimer’s disease | *APP, PSEN1, PSEN2, S100A9, CR1, BIN1, TREM2, CLU, CTNNA3, DNMBP, SORL1, BACE1, PICALM, GAB2, LPR6, ADAM10, ABCA7, CD33, TOMM40* |
| Amyotrophic Lateral Sclerosis (ALS) and Frontotemporal dementia (FTD) | TDP43, CHMP2B, SIGMAR1, VCP, FUS, GRN, MAPT, UBQLN2, ALS2, TAF15, FIG4, OPTN, DAO, HNRNPA1, SOD1, ANG, VAPB, SQSTM |
| Dementia with Lewy Bodies | PINK1, PARK7, PARK9, GBA, SNCA, PRKN, LRRK2 |
| Other neurodegenerative | SPAST, CYP7B1, SPG11, CSF1R, NOTCH3, PRNP |
